# Tunable Graphene/Nitrocellulose Temperature Alarm Sensors

**DOI:** 10.1021/acsami.2c02340

**Published:** 2022-03-11

**Authors:** Wenyuan Wei, Yangpeiqi Yi, Jun Song, Xiaogang Chen, Jinhua Li, Jiashen Li

**Affiliations:** † Department of Materials, The University of Manchester, Manchester M13 9PL, U.K.; ‡ Hubei Key Laboratory of Polymer Materials, School of Materials Science and Engineering, Hubei University, Wuhan 430062, China

**Keywords:** graphene, composite materials, nitrocellulose, fire alarm, temperature sensors, warning response

## Abstract

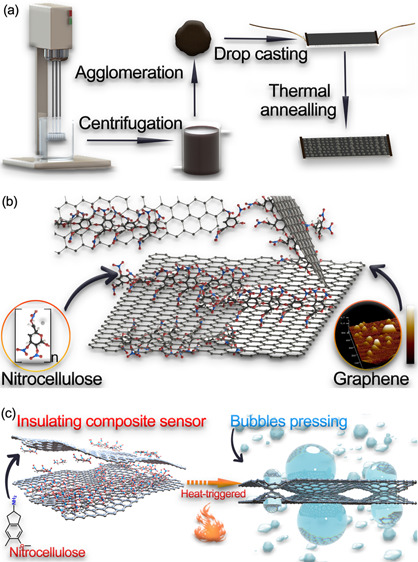

Tunable temperature alarm sensors were prepared using multilayer graphene and nitrocellulose (NC) to reliably monitor early high temperature risks. The graphene/NC alarm sensor keeps in a state of electrical insulation, however, turns electrically conductive at high temperatures, such as encountering a flame attack. Its response time is limited to only a few seconds because of a quick chemical reaction of NC. The 90% graphene/NC (wt % ratio 1:9) composite alarm sensor stably remains insulated at an ambient temperature of 200 °C, resulting in a satisfactory responsive temperature (232 °C), instant response time (4.4 s), and sustained working time in the flame below the ignition temperature of most combustibles. Furthermore, the response temperature and time of the alarm sensor can be tuned by graphene/NC ratios to reduce the fire risk of various combustible materials in different fire-prone scenarios and thus has promising applications in both indoor and outdoor environments. The sensor has also proven to work in the form of paint, wallpaper, and other composites due to its superior flame retardancy property, as well as under extreme conditions (i.e., underwater and vacuum).

## 1 Introduction

There were approximately 167 000 fires in the UK from 2017 to 2018.^1^ Once a fire breaks out, the disaster not only causes huge economic losses but also endangers lives. Driving the development of fire safety strategies, especially preventative methods such as early fire detection that helps to avert severe damage before it occurs is, therefore essential in ensuring the welfare of lives and property. Among various existing devices, smoke detectors and infrared heat detectors are the primary fire sensors that are widely utilized indoors.^2^ However, as both of them are activated by smoke, which is only emitted after the flaming combustion has already begun, the response time of these traditional detectors usually exceeds 100 s.^3^ The delayed response of these detectors makes it almost impossible to provide timely and effective warning signals to reduce losses of life and property. To this end, developing an early-warning fire detection system with a faster response time is of critical importance. A severe fire accident usually initiates with the induction of a temperature increase in some combustible material by an external heat source, leading to its endothermic pyrolysis.^4^ This causes the vaporization of decomposition products and thus generates a further rapid increase of temperature, triggering the fire as a result. Consequently, an early-warning fire detector with an improved response speed would facilitate safety, but only if it also complies with the following requirements: it must provide warning signals for abnormally high ambient temperatures, and it must maintain structural stability during a flame attack.

Existing research indicated that graphene and graphene oxide (GO) can be used as effective materials for fire alarm sensors due to their high electron mobility, superior thermal conductivity,^5^ high mechanical properties,^6^ and structural stability under high temperatures. Researchers took advantage of the electrical insulating features of GO that would be reduced and thus turn conductive in a high temperature environment and produced a series of temperature sensors. These sensors demonstrate a lower thermal response temperature and faster response behavior when compared to traditional fire detection methods which theoretically reduce the response time from 100 to 2–6 s.^7−11^ Chen et al.^8^ prepared a fire alarm using fire-resistant inorganic paper as a coating material based on ultralong hydroxyapatite nanowires and GO. It was arranged with an electrical connection between the composite paper, an alarm lamp, and an alarm buzzer to achieve an instant response time (less than 2 s) under a relatively high experimental temperature. Similar morphology designs have also been successfully utilized by other researchers, who employed different materials in conjunction with GO. Endeavoring to enable fire prevention through detecting high environmental temperatures, that are, meanwhile, below the ignition temperature of combustibles, Wu et al.^9^ prepared hierarchical coatings on GO and silicone. These coatings were applied to different combustible substrates to facilitate the exhibition of distinct temperature-responsive electrical resistance changes, creating a highly effective early-warning sensor. However, the authors did not define the accurate response temperature or time of their sensors, which is relevant and necessary information for safety and sensor design as the GO/silicone composite is coated directly onto a combustible material. Other researchers grafted silane on GO to manufacture flexible flame-retardant paper.^12^ Similar to the work of Yu et al.,^13^ the insulating silane–GO paper could be thermally reduced to form a conductive network in a high-temperature environment (e.g., in direct exposure to fire). Xu et al.^11^ synthesized rectangle-shaped GO wide-ribbon (GOWR) sheets from carbon nanofoams and fabricated the GOWR-wrapped melamine–formaldehyde sponge through dip-coat processing. However, such a sponge only started to respond at around 450 °C, which was much higher than the desired response temperature.

It is worth noting that the aforementioned studies focused only on utilizing the mechanism of GO reduction to achieve transformation of the conductive state, not on other graphene-like materials. Herein, the electrical conductivity of graphene and the insulation of nitrocellulose are integrated to prepare a new fire alarm sensor. The excellent properties, such as high electron mobility^15,16^ and superior thermal conductivity, have introduced graphene as the optimal candidate for temperature sensor applications at the micron-scale or even at the nanoscale.^18^ Given the method for preparing graphene with liquid-phase exfoliation,^19−23^ various polymers have been proven to be effective in producing uniform graphene dispersion as a dispersant, which contributes a simple strategy to manufacture graphene/polymer composites effectively. Nitrocellulose (NC), one kind of chemically modified cellulose, has recently been proven to be a graphene-dispersing polymer that effectively disperses graphene in organic solvents. NC, due to its unique characteristics in instantaneous pyrolysis and oxygen-free combustion, provides an inspiration for its potential in the design of a temperature-sensitive early-warning sensor. So far as we know, no substantial research has reported any use of graphene and NC in temperature alarm sensors. This study innovatively presents a graphene/NC early-warning fire sensor that efficiently responds to temperature changes and avoids severe damage. Although the graphene/NC membrane remains electrically insulated normally, it turns conductive at high temperatures and therefore can be used in sensors of an alarm system. Furthermore, the response temperature and sensitivity of these sensors can be tuned by adjusting the ratio of graphene and NC to react in different fire-prone scenarios (each 1% increase in NC content leads to a ∼1.8 °C increase in the response temperature). Meanwhile, the tunable temperature sensor has proven to withstand extreme conditions such as underwater or vacuum environment as thermal degradation of NC can take place in oxygen-free environments. The flame retardancy test indicates the sensor’s superior and stable function when employed on a solid surface (see Supporting Information Figure S1 and Movies S1, S2, and S3). All types of graphene/NC sensors with various NC content provided continuous danger alarm even when the samples were removed from a flame or specific temperature environment, demonstrating effective and stable fire warning for detecting high fire risk of combustible materials for applications in various scenarios.^24^

## 2 Experimental Section

### 2.1 Materials

Collodion solution (4–8% NC in ethanol/diethyl ether), ethyl acetate, acetone, and *n*-butyl acetate (ACS Reagent grade) were purchased from Sigma-Aldrich. Natural graphite flakes (−10 mesh, 99.9%, metals basis) were purchased from Alfa Aesar. Sodium chloride (99.99%, metals basis) was purchased from Sigma-Aldrich and used to induce the flocculation of graphene.

### 2.2 Preparation of Graphene

Graphene was manufactured from natural graphite flakes by the liquid exfoliation method. First, the as-received collodion solution was poured into a Petri dish and evaporated in a fume hood overnight until no change in weight was observed. Dried NC (10 g) was cut into small pieces and added into a 1000 mL mixed solution in a ratio of 1:4 ethyl acetate and acetone with natural graphite flakes (100 g). With a water-cooling system, the mixture was operated in a high shear mixer (Silverson L4RT) for 2.5 h at 6000 rpm and produced a graphene/NC solution that was then centrifuged at 3000 rpm for 30 min (three times) to remove the large graphite flakes. The supernatant was collected and then added with a 40 g L^–1^ aqueous salt solution to induce the flocculation of graphene and NC. After that, the sediment was harvested and left to dry at 70 °C in an oven. Once the organic solvent was completely evaporated, the graphene/NC flocculation was rinsed with deionized water several times until the surplus sodium chloride was removed. The resulting solid was dried and stored in bottles prior to subsequent use.

### 2.3 Fabrication of the Composite

The graphene/NC mixture was then dispersed directly in *n*-butyl acetate at a concentration of 10 or 30 mg mL^–1^. Additional NC was added into the suspension to gain different ratios of NC in the composite. With the abovementioned methods and the original suspension, the first fabricated composite where NC content was 60% was marked G@NC60. Extra NC was added to the original suspension to produce different NC content composites such as G@NC75 and G@NC90. Altogether, three types of samples, where NC content was 60, 75, and 90%, respectively, were produced and employed in the current study. The solution was then drop-casted onto a pre-cleaned blank glass slide (7.5 cm long and 2.5 cm wide) and evaporated under ambient conditions, leaving a coating membrane of graphene/NC. The thickness of these films can be controlled by the amount of solution added to the substrate. Two edges of the membrane were connected to copper wires as external electrodes for further tests of resistance change.

### 2.4 Characterizations

To investigate the chemical changes of the graphene films after the thermal treatment, thermogravimetric analysis (TGA), Raman spectroscopy, atomic force microscopy (AFM) analysis, and Fourier transform infrared (FTIR) spectroscopy were employed. The samples utilized for all the abovementioned characterization methods were divided into two types: (i) pre-annealing G@NC films and (ii) G@NC samples after thermal treatment at 260 °C. FTIR data were obtained using an FTIR spectrometer (Nicolet 5700, Nicolet Instrument Company) between 400 and 4000 cm^–1^. Raman spectra of various samples before and after thermal treatment were acquired from 200 to 4000 cm^–1^ using a Renishaw System 1000 Raman spectrometer at ×50 objective, with an incident power of 2.3 mW. AFM images of graphene sheets were recorded with a Bruker Dimension Icon in peak-force mode. The spectral resolution of system was within 1.5 cm^–1^ at 514 nm. TGA data of various samples were generated using the TA Instruments Q500 in an air or nitrogen atmosphere at a heating rate of 10 °C min^–1^ from room temperature to 800 °C. Scanning electron microscopy (SEM) images were collected by a scanning electron microscope (Carl Zeiss AG-ULTRA 55). Tensile tests were performed using an Instron single column table frames model 3344 (100 N load cell) at a speed of 1.0 mm min^–1^ at room temperature following the ASTM standard 882, and the sample thickness was measured by SEM.

Electrical resistance variation of alarm sensors with different NC content was monitored utilizing the two-electrode method, and two opposite edges of the samples were affixed to copper wires as electrodes with the help of a copper sheet. The response temperature tests were performed using an oven (Carbolite LHT 4/30) that allows wires to pass through when heating up to measure the temperature and resistance relationship at various heating rates such as 2.5 °C, 5 °C, and 7.5 °C min^–1^. A custom-made LabVIEW program controlled the heating rate of the oven, and the sensor data was acquired from a multimeter (Gw Instek GDM-8342) simultaneously between room temperature and 260 °C. Other response time tests were performed on various specimens which were instantly placed in the same oven as stated above, and before testing, the environment temperature in the oven was set as 100, 200, 300, and 400 °C, respectively. A vertical flame test was also performed to investigate the response time of sensors with various NC content in a simulated real fire scene. The fire from an alcohol burner was applied on the sensor samples connected with the multimeter and flash alarm. The additional tests are conducted to evaluate the operating state of the alarm sensor underwater. The response time was recorded using a camera, and the data reported in this work were the averages of three experiments.

## 3 Results and Discussion

### 3.1 Fabrication, Structure, and Physical Properties of Graphene/NC Composite

**Figure 1:**
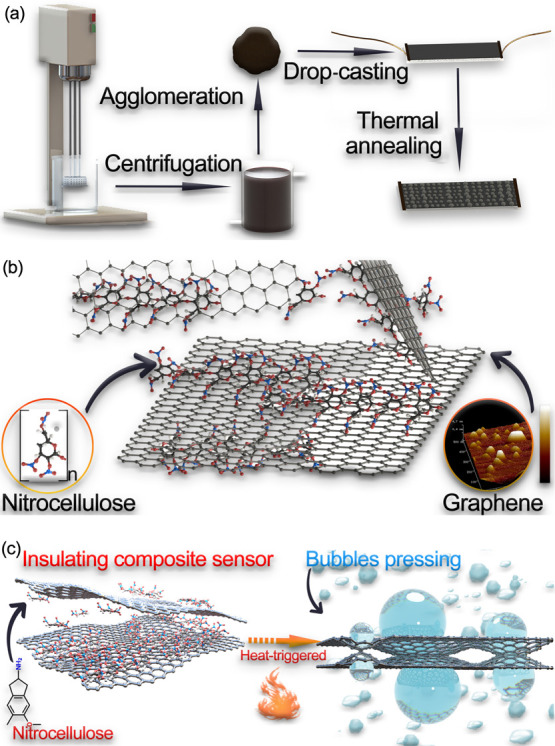
Schematic illustration of the (a) fabrication process of graphene/NC composite alarm, (b) chemical structure of NC and molecular structure of graphene/NC composite, and (c) schematic illustration of temperature-induced sensitive resistance transition of graphene/NC composite alarm used to detect a high fire risk in a real fire scene.

We employed a facile and time-saving method, liquid exfoliation, to prepare the graphene from graphite. Figure 1a illustrates the fabrication process of graphene clearly. A prevalent strategy for enhancement of the stability of graphene in organic solvents is to employ a stabilizing polymer. Here, NC as an effective stabilizing dispersant, coated and insulated the graphene sheet. Pristine graphene was exfoliated in a scalable manner by shear mixing of graphite in a solution of NC, acetone, and ethyl acetate. Unexfoliated graphite flakes were removed by centrifugation, yielding a stable dispersion of few-layer graphene with a concentration as high as 1 mg mL^–1^, which is not high enough to cast a uniform film. Though graphene is dispersed in the solution, the non-spherical graphene sheets are difficult to flow on the surface of a substrate. As the solvent used in the mixed solution, ethyl acetate and acetone enjoy strong volatility resulting in graphene and NC particles moving slowly toward the liquid-coated outer layer by evaporation during the evaporation of the organic solvent. The drop-cast composite membrane suffered from the influence of the coffee ring effect when using the relatively low concentration of graphene/NC solution because graphene particles have a large amplitude fluctuation on the air–liquid interface layer. As the organic solvent decreases during evaporation, the flow of graphene particles might cause clogging. Therefore, it is necessary to increase the concentration and viscosity of the graphene dispersion to fabricate a uniform and smooth composite membrane.

Sodium chloride solution was added here to the dispersion to induce flocculation of the graphene/NC composite. Graphene flakes or particles consist of sp^2^ carbon atoms arranged in a planar two-dimensional structure. The electron distribution has π-orbitals on two sides of the graphene plane which represent electron densities available to the molecule. The graphene sheets are negatively charged with a superb store of electrons. As an electrolyte, sodium chloride is ionized in water, and the cations (positively charged sodium ions) can coagulate the negatively charged graphene sheets. Additionally, flocculated graphene sheets are coagulated into a solid porous structure with a certain mechanical strength due to the presence of NC. Concentration of the re-dispersed graphene/NC dispersion is adjustable whereas with its highest concentration can reach 60–70 mg mL^–1^ (NC included), yet is limited by the viscosity of the whole dispersion.

### 3.2 Thermogravimetric Analysis, Raman Spectroscopy, and FTIR Spectroscopy

**Figure 2:**
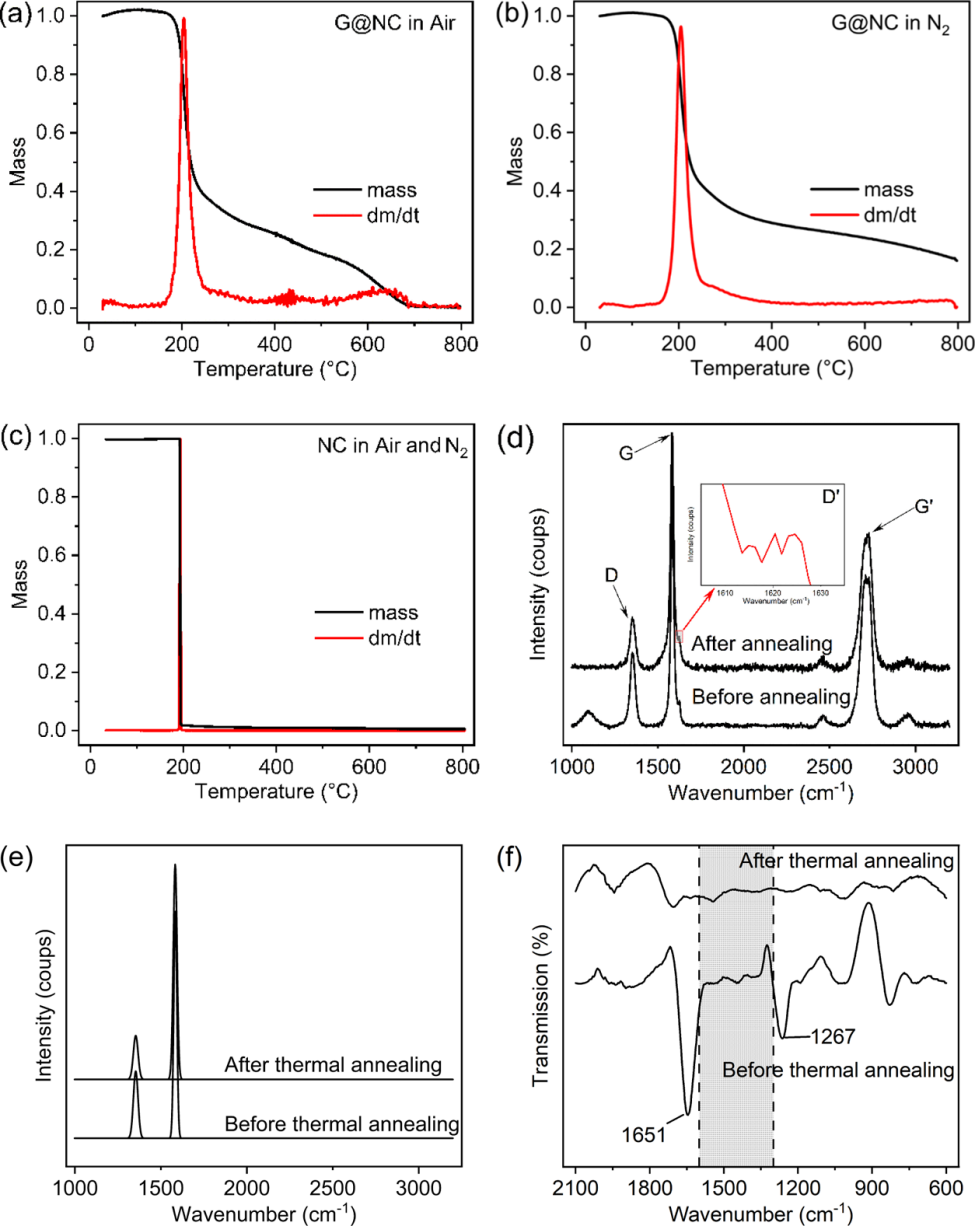
TGA curves showing mass and mass derivative for (a) graphene/NC in air, (b) graphene/NC in nitrogen, and (c) pristine NC. Raman spectra of (d) graphene/NC films after and before thermal treatment and (e) peak fitting, and (f) FTIR characterization of graphene/NC composite before and after thermal treatment.

TGA was carried out in an air or nitrogen atmosphere from 30 to 800 °C at a temperature ramp of 10 °C min^–1^. As shown in Figure 2a, TGA curve indicates two primary loss peaks in the mass derivative curve corresponding to NC at ∼200 °C and graphene at ∼580 °C, respectively. For the fully nitrated NC, the combustion reaction formula can be presented as
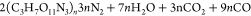


In comparison with the pristine NC completely degraded at ∼200 °C, the graphene/NC composite demonstrated a downward trend in curve after the first weight drop peak at a similar temperature. This difference indicates that the interaction of graphene and NC alters the decomposition characteristics of the polymer and prevents or delays the complete thermal degradation of NC in the composite at ∼200 °C because pristine NC should remain only as gases after the thermal decomposition as shown in the formula above. Meanwhile, another mass loss in air observed at ∼620 °C is consistent with the characteristics of graphene decomposition between 550 and 650 °C. Compared to the TGA curve shown in Figure 2a, graphene/NC composite sample treated in nitrogen did not show any significant mass loss peaks after 200 °C, as no decomposition reaction occurred in graphene and NC residual amorphous carbon in the absence of oxygen. Although the volatile decomposition products released from rapid NC combustion facilitated the formation of porous microstructures, it is apparent that a fraction of the polymer residues were not completely decomposed. One possible explanation is the dependence of NC decomposition on the heating rate. Slow or fast annealing results in the controlled release of decomposition products due to the chemical or physical interaction between NC and graphene. Results obtained from TGA divulge that 60% residue remained at ∼200 °C approximately after NC decomposition including graphene and NC polymer residues. Because the downtrend of TGA data obtained in air and nitrogen is nearly identical after 200 °C, it could be estimated that the ratio of graphene to NC in solution before flocculation, however, after liquid-phase stripping is 2:3 approximately.

A small and sharp peak associated with NC at ∼198 °C on the TGA curve for G@NC was found to exist but difficult to observe. As decomposition of NC is not typically carried out under mild conditions, it inevitably generated an obvious gas recoil at the instant moment of deflagration. In contrast, the slight mass gain was not observed in the pristine NC TGA curve, which might be explained by the presence of graphene that imposed restrictions on the escape of decomposition residue through physical or chemical interactions of NC and graphene. When the graphene/NC composite was heated to 198 °C, it is most likely that NC deflagration gas was trapped between the graphene sheets and released gradually from the film. Meanwhile, compared to graphene/NC composite, pristine NC film did not reflect any mass loss peak which was attributed to the absence of the sturdy graphene network membrane to catch the gas released by the instant combustion of NC. When instantaneously released gases were trapped by graphene, reactive force of the gas recoil caused the instantaneous mass increase.

Raman spectroscopy has become an ideal analytical technique for detecting the evolution of defects and quantifying the disorder of graphene due to its high structural selectivity and high spectral and spatial resolution. Raman spectra of the graphene/NC composite after and before thermal treatment exhibited several prominent peaks involving two typical bands at 1351 cm^–1^ (D-band) and 1583 cm^–1^ (G band) analogously. The defect density in graphene is characterized by the relationship between the defect-related peak (*D* peak) and the main characteristic peak caused by in-plane vibration of carbon atoms (*G* peak). In general, both the ratio of peak height (*D*/*G*) and peak area (ID/IG) can estimate the quality of graphene. As Figure 2d,e reported, both *D*/*G* and ID/IG ratios increased after thermal treatment at 260 °C. Lower ratio generally signifies the fewer defects in graphene, which can be attributed to the high sp^2^-content amorphous carbon produced by the decomposition of NC as mentioned in TGA section before. Meanwhile, compared to the heated samples, the graphene/NC composite, before receiving thermal treatment, demonstrated an additional peak in the band around 1090 cm^–1^, which is roughly consistent with the typical NC Raman spectrum peak. In addition, both two types of samples have a defect-induced *D*′ shoulder peak at around 1620 cm^–1^. Regarding the number of graphene layers, it is apparent that graphene prepared with the current method has more than four layers as the *G*′ peak is smaller than the *G* peak. However, the layer number depends on the choice of solution and machine for liquid-phase exfoliation.

Although Raman spectroscopy provides an effective analysis of graphene, using FTIR spectroscopy was also necessary to investigate chemical properties of graphene/NC composites before and after thermal treatment. Generally, the first stage of NC decomposition is the denitration process. In the annealed graphene/NC composite samples, as NC was decomposed, the peaks related to nitrate functional groups lose intensity. Therefore, for thermal treatment below 300 °C, although NC was not completely decomposed, the peaks at 1651 and 1267 cm^–1^ related to nitrate functionality are not measurable. This observation is consistent with the previous literature indicating the characteristic of NC denitration and decomposition at around 200 °C.^25^ Moreover, several prominent peaks in the range of 1300–1600 cm^–1^, indicating the covalent bonding character as shown in the shaded area in Figure 2f, can be clearly observed in the sample before thermal annealing. The small peaks at ∼1380 and ∼1250 cm^–1^, which disappeared after annealing due to the increase of the resulting molecular symmetry leading to a decline in the vibration intensity, are consistent with the skeletal C–C modes of high sp^2^-content amorphous carbon.^26^

### 3.3 Scanning Electron Microscopy

**Figure 3:**
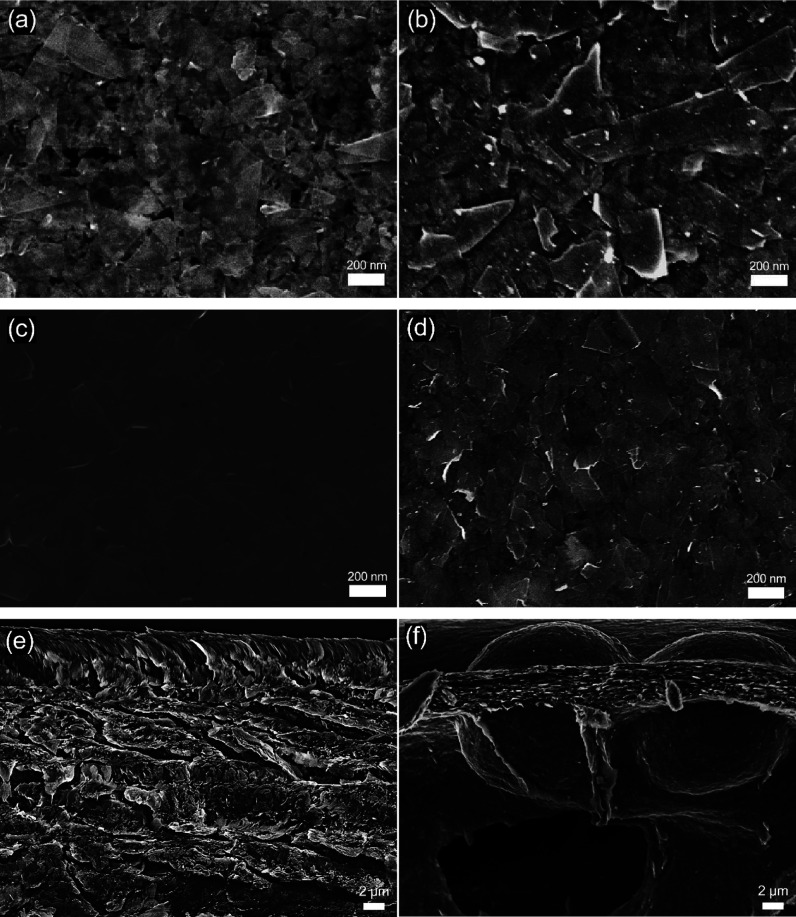
Typical SEM images in top view of (a) 10 mg mL^–1^ before thermal treatment, (b) 10 mg mL^–1^ after thermal treatment, (c) 30 mg mL^–1^ before thermal treatment, and (d) 30 mg mL^–1^ after thermal treatment. Cross-sectional images of (e) 30 mg mL^–1^ before thermal treatment and (f) 30 mg mL^–1^ after thermal treatment.

The microstructure of the composite membrane is expected to impact its performance characteristics. Theoretically, a dense, close-connected graphene network can exhibit excellent mechanical and electrical performance. The microstructures of the films drop-cast before and after thermal treatment with different samples of graphene/NC solution are compared to evaluate the influence of concentrations. Before thermal treatment, as Figure 3a,c reveals, a dense and smooth surface was formed by a film drop-cast with a concentration of 30 mg mL^–1^ whereas the 10 mg mL^–1^ sample failed to form a continuous smooth surface due to an inadequate content of NC. After thermal treatment, as shown in Figure 3b,d, a high degree of graphene flakes alignment in the plane of the film drop-cast with a concentration of 30 mg mL^–1^ has been clearly observed, which efficiently avoided the flake-to-flake overlap. Regarding the drop-cast film of 10 mg mL^–1^ solution, graphene flakes were further apart in space, resulting in a sparse network structure that negatively affected the efficiency of charge transfer. Therefore, a high concentration of graphene/NC dispersion (e.g., 30 mg mL^–1^) is crucial for excellent electrical conductivity as well as for the rate of resistance variation, which is of great implication for fire alarm preparations.

The cross sections of the film distinctly presented the influence of NC on the electrical conductivity and basic working mechanism of the alarm sensor before and after thermal treatment. Wrapping the graphene flakes tightly before thermal treatment (see Figure 3e) and the insulating polymer NC impeded charge transfer between graphene flakes. In the heated film, NC was decomposed and released a large amount of gas to form bubbles both inside and on the surface of film (Figure 3f). The original insulated obstruction disappeared, whereas on the other side, the generated bubbles pushed the previously dispersed graphene flakes into interconnections. Therefore, a good connection between the graphene flakes resulted in the electrical and mechanical connection of graphene/NC composite being restored again. The energy-dispersive X-ray spectroscopy (EDS) data shown in Figure S2 (Supporting Information) evidently proves that the nitro group disappeared after thermal treatment.

### 3.4 Mechanical Properties

**Figure 4:**
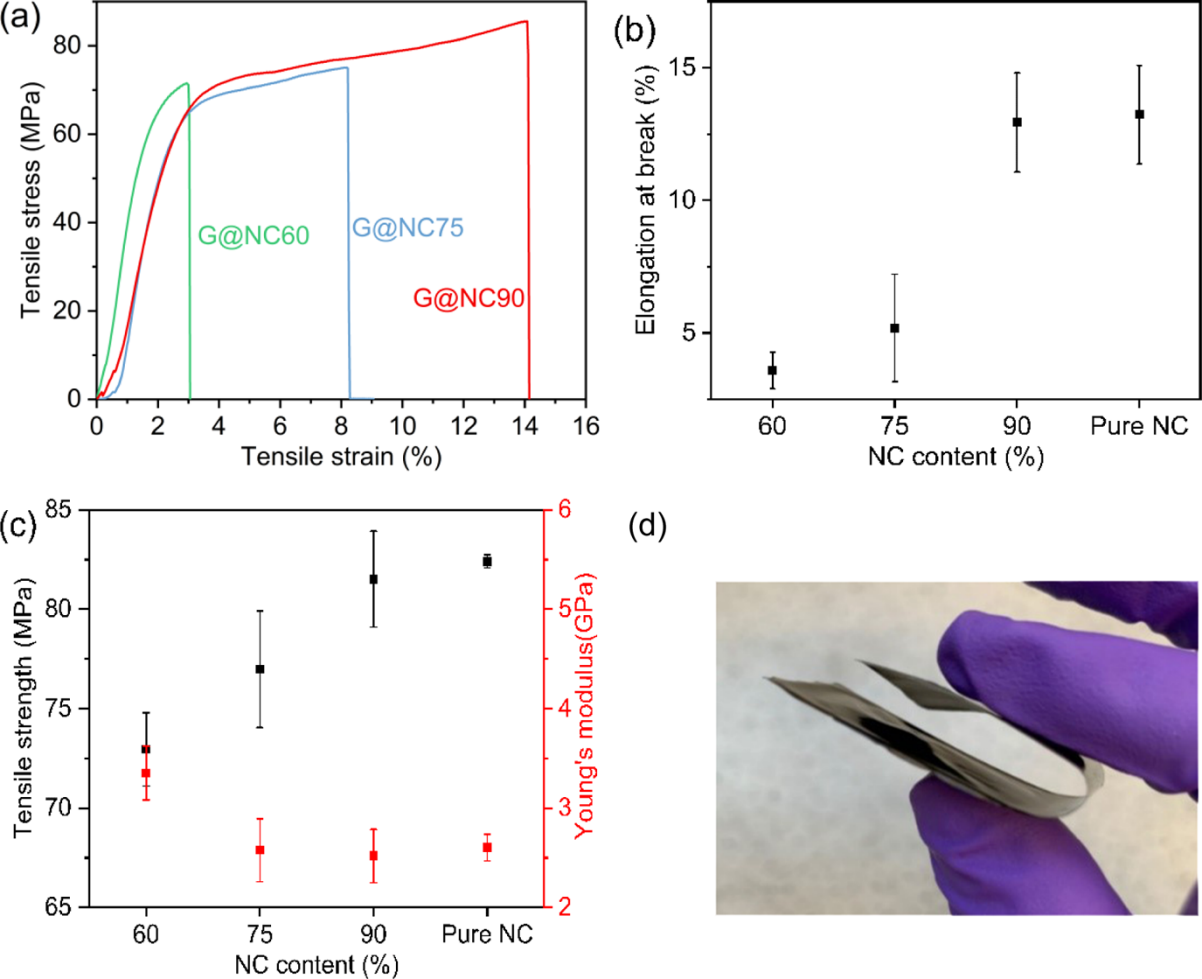
Influence of NC content on tensile properties of graphene–matrix composites. (a) Typical stress–strain curves, (b) elongation at break, (c) tensile strength for sensors with different NC content, and (d) photograph showing the flexibility of graphene/NC sensor.

Mechanical properties of the graphene/NC composite films depend not only on fibril modulus but also on orientation and degree of interaction between NC and graphene within the film. Figure 4 below summarizes the stress–strain curves of pure NC and graphene/NC composites, and the film’s mechanical properties such as tensile elongation at break and tensile modulus of elasticity. Data obtained by ASTM standard 882 with relatively less error showed the effective use of slow evaporating organic solvents in reducing the coffee ring effect (Table S1, see Supporting Information). Figure 4a indicates typical strain–stress curves and Figure 4b highlights the tensile modulus and tensile strength of graphene/NC composite films with different NC contents. Obviously, the tensile strength decreased with an increasing graphene content, whereas the tensile modulus was enhanced comparing with the pristine NC film. At 90% NC content, the graphene/NC composite films shared similar tensile modulus and strength with pure NC film. Meanwhile, it could be concluded from Figure 4c, that the elongation at break of these composites also displayed a similar trend to the tensile strength. As previous studies showed, trace amounts of graphene enhanced the mechanical properties of cellulose and graphene composites. The above results indicated that the large amount of graphene does not have a significant impact on the tensile properties, where the film can still present a flexible state (Figure 4d).

### 3.5 Resistance Change and Working Mechanism

The mechanism of temperature response, as shown in Figure 1c, is based on the thermal degradation of NC in short. The SEM image (Figure 3e) and Figure 1c show the working principles of the graphene/NC alarm sensor. The NC before thermal treatment tightly wrapped the graphene flakes, and this insulating polymer impedes charge transfer between graphene flakes. Meanwhile, the NC in heated film decomposed and released a large amount of gas to form bubbles both inside and on the surface of the graphene/NC film (Figure 3f). The existing insulated obstruction disappeared, and on the other side, the generated bubbles pushed the previously dispersed graphene flakes into interconnection. Therefore, the excellent connection between the graphene flakes results in the electrical and mechanical connection of the graphene/NC composite being restored again and instantly. To sum up, the alarm sensor works perfectly as long as the thermal degradation of NC in the composite can successfully occur at the corresponding temperature.

The interaction between graphene and NC is crucial for studying the detailed mechanism of temperature response. The bonding between graphene and NC after annealing is likely to be regarded as the covalent bonding character such as skeletal C–C modes of high sp^2^-content amorphous carbon according to the peaks shown in FTIR spectroscopy results. In comparison with the pristine NC completely degraded at ∼200 °C, the graphene/NC composite demonstrated a downward trend in the curve after the first weight drop peak at a similar temperature. This difference indicates that the interaction of graphene and NC alters the decomposition characteristics of the polymer and prevents or delays the complete thermal degradation of NC in the composite at ∼200 °C because pristine NC should remain only as gases after the thermal decomposition. This is also the reason why the response temperature of the graphene/NC alarm sensor is called “Tunable”. In addition, temperature changes strongly influence the thermal motion in graphene, and the increase in temperature can reduce resistivity. For graphene prepared by liquid-phase exfoliation in this study, multilayer graphene (MLG) has a negative temperature coefficient of resistance (TCR) value due to no electric field intensity existing in the interlayers, which will be discussed later.^27^ However, the characteristic that graphene resistance decreases with temperature has no effect on the working principle of the sensor alarm.

An efficient alarm sensor for early-fire warning has to offer an alarm signal at high environmental temperature with extremely fast response time and maintain structural stability throughout the flame attack. Although most polymeric materials ignite around 300–500 °C, the majority of commercial temperature-sensitive fire alarms only respond between 60 and 80 °C which is much lower than the ignition temperature. This may be explained by the fact that the flame temperature of the combustible materials is much higher than the average temperature at each point in the burning space. When a fire breaks out in a compartment, the rise of the hot air flowing results in the formation of a higher temperature layer of hot air above the compartment whereas the fire temperature commonly refers to the temperature of these hot air layers. In most cases, however, the indoor fire alarm is installed on the ceiling of the compartment, leaving it less functional as its fire response temperature is much lower than the environmental temperature around combustible materials. Prior to detecting the extensive fire and sending the alarm signals, commercial temperature-sensitive fire alarms are not able to initiate early-fire warning in the early stage of fire as in the growth phase (the first stage) of the idealized fire temperature curve shown by Milan et al.^28^ Unlike the traditional fire alarm, the graphene/NC temperature alarm sensor meets all the requirements of efficient fire detection: (i) extraordinary rapid response and steady performance, (ii) structural stability during flame attack, and (iii) early warning signal for abnormally high environmental temperature.

**Figure 5:**
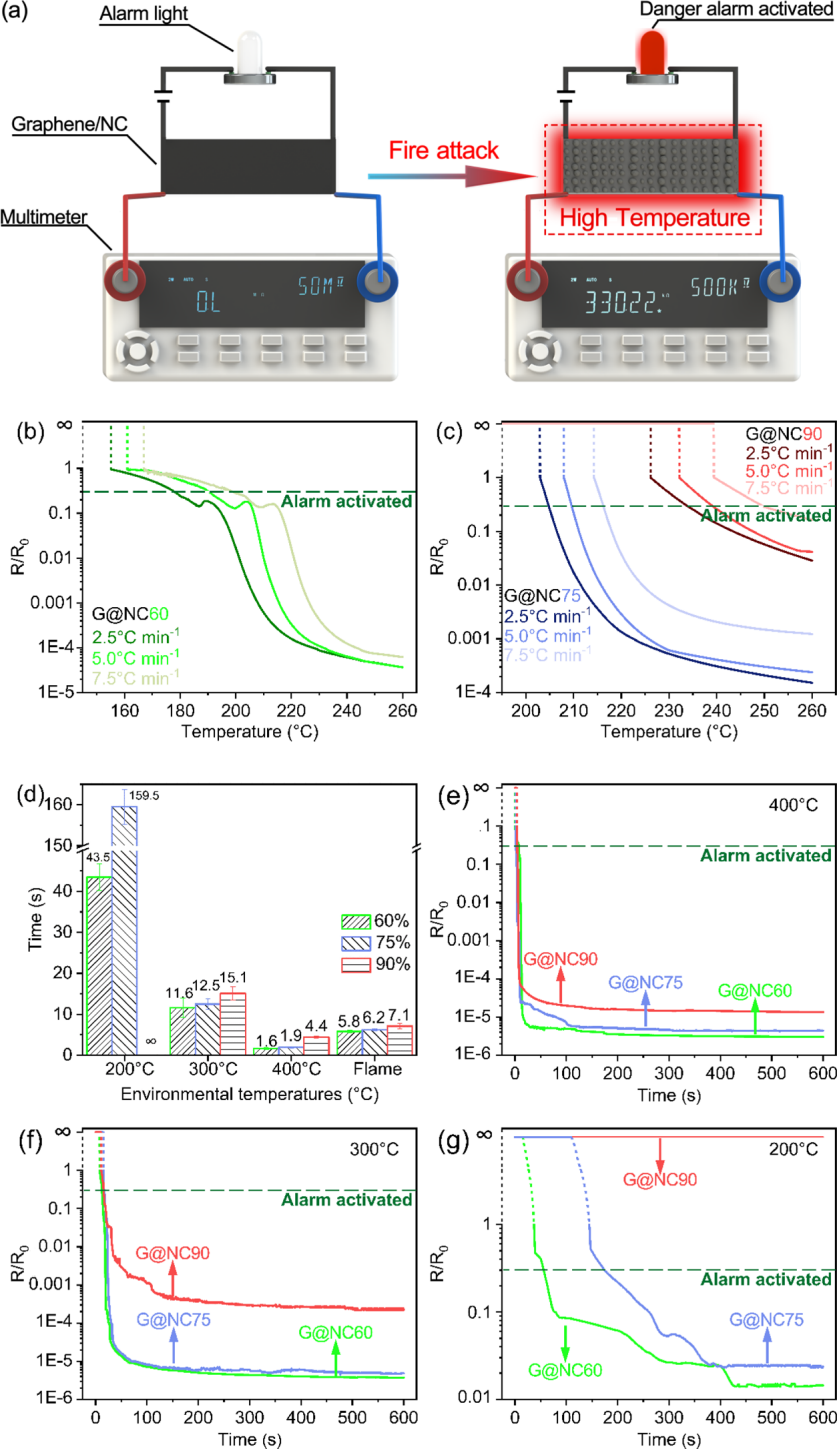
Flame rapid detection and fire alarm of graphene/NC composite alarm sensors. (a) Schematic illustration of flame detection processes using the alcohol burner, (b) electrical resistance change of G@NC60 at various heating rates from 30 to 260 °C, (c) electrical resistance change of G@NC75 and G@NC90 at various heating rates from 30 to 260 °C, and (d) response time under different NC content and heating rates. Resistance change observed at different environmental temperatures: (e) 400, (f) 300, and (g) 200 °C.

The instant response process of the graphene/NC alarm sensor for fire detection depends on the rapid change in electrical resistance, which is in situ reduced sharply at specific temperatures. To investigate the response temperature of alarm sensors at different heating rates, the resistance change of these composites with different NC contents was measured using an oven with controlled heating rates (2.5, 5, and 7.5 °C min^–1^). It should be noted that because the graphene/NC alarm sensors were originally non-conductive before being heated, the initial resistance (*R*_0_) was set as the maximum measuring range (5.1 × 10^7^ Ω) of the multimeter. As depicted in Figure 5b,c, the response temperatures were strongly dependent on their ratios of NC in the composite, that higher NC content led to higher response temperature. The light-emitting diode (LED) warning light can be triggered to illumine when the resistance decreased by approximately 1 order of magnitude (as shown by the dotted line in Figure 5b,c). The response temperature of G@NC60 heated at 2.5, 5, and 7.5 °C min^–1^ was indicated as 155, 161, and 167 °C as shown in Figure 5b, respectively, showing each increase of 2.5 °C min^–1^ heating rate resulting in an increase of ∼6 °C in response temperature. Similar trends were also observed in G@NC75 as well as G@NC90 (Figure 5c). Meanwhile, examining the above results, it could be concluded from the alarm sensor’s invariability (which was proved by temperature-resistance curves of samples with various NC contents) that, the integrated alarm sensor device based on the graphene/NC composite could provide a stable and reliable signal of early-fire warning to reduce the high fire risk in various fire-prone scenarios.

Considering that the oven-set tests were not directly connected to the alarm system, the dashed lines for alarm activation as shown in Figure 5b,c could only be concerned as a reference. Therefore, the dramatic transition displayed here by the electrical resistance values of G@NC60 can be utilized as an application of the rapid response behavior of the graphene/NC alarm sensor. As presented in Figure 5b, G@NC60 first experienced a cliff fall in resistance at ∼155 °C heated under a temperature ramping rate of 2.5 °C min^–1^. The electrical resistance curve then displayed a steady decline until ∼185 °C after that extreme descent, which is not visible in that of G@NC75 and G@NC90. It was as well interesting to note that a small resistance rise peak appeared at 190 °C, which was followed by the second rapid downward transition. The possible explanation is that the violent thermal decomposition of G@NC60 resulted in a deformation of the film in the early stage of pyrolysis. As discussed earlier, the volatile decomposition products of NC contain a large amount of gas, leading to bubbles formed on the surface of graphene film. The existence of this capability has been confirmed in the discussion parts of FTIR data and “a sharp and small peak” in TGA. The formation of bubbles depends on the membrane’s ability to capture gas. For sensor alarms with high graphene content, such as G@NC60, the reaction is also more intense due to the fast speed of meeting the requirement of sufficient heat absorption to generate pyrolysis. Therefore, the bubbles produced in the thermal degradation process are generally large and numerous, resulting in a large film deformation due to the capture of gas. This deformation is mainly responsible for a temporary rise in resistance. On the contrary, for sensor alarms with relatively low graphene content, such as G@NC75 and G@NC95, the high-temperature response and milder thermal degradation process result in the formation of small and dense bubbles. Hence, a resultant small deformation will not affect the resistance curve.

Moreover, we found that the graphene/NC composites with different NC contents correspond to different response temperature. G@NC75 and G@NC90 demonstrated completely different temperature-resistance curves compared to that of G@NC60 in that only one drastic transition of electric resistance was observed as shown in Figure 5c. As expected, based on the statistics illustrated above, the response temperature of graphene/NC alarm sensors was projected to a certain regular climb with the increase of NC contents, which means that the response temperature of this sensor alarm can be controlled by adjusting the NC contents to realize its applications in different fire-prone scenarios. In addition, the oven was turned off immediately when the temperature reached 260 °C with samples left inside the oven for 600 s to estimate stability of the electrical resistance. All three types of NC content samples produced lasting alarm signals even after the removal of heating source as shown in Figure S3. However, the judgment of alarm response might be affected by the NC’s heat absorption behavior in the sensors. Thus, the accurate response time should be determined during direct flame attack tests and fixed-temperature heating in the oven.

Response time is one of the most critical characters in a fire warning device. To evaluate the response time of graphene/NC alarm sensors more accurately and to investigate the impact of ambient temperature on alarm response quantitatively, direct flame attack and different environmental temperatures of 100, 200, 300, and 400 °C in the oven were employed in this study. To simulate the actual working conditions during a flame attack, the alarm sensors with different NC contents (60, 75, and 90%) were set under the same testing setup as shown in Figure S4a. The detection processes of graphene/NC alarm sensors have been illustrated in Figure 5a (see also Figure S5, Movies S5, S6, and S7Supporting Information for more details). Considering that the ignition temperatures of most combustible polymeric materials range from 300 to 500 °C, which is similar to the inner and outer flame temperature of the alcohol lamp, this test environment can effectively simulate the applications of the graphene/NC alarm sensor in flame rapid detection and early warning sensors. Figure 5d shows the alarm response times (to illuminate LED lights) of graphene/NC alarm sensors with different NC content at different ambient temperatures and direct flame attacks. Obviously, the resistance of all samples with different NC contents declined drastically within the applied time, and higher ambient temperature led to more remarkable resistance changes as well as faster alarm response time. The resistances of all three NC content samples barely changed at 100 °C, indicating that these graphene/NC alarm sensors have outstanding reliability, regardless of their NC contents, for potential applications at ambient temperature. The fact that G@NC90 maintained stability at 200 °C suggested that alarm sensors with different NC contents can respond to fire scenarios at different ambient temperatures. Observing Figure 5e,f, it was clear that except for G@NC90, G@NC60 and G@NC75 exhibited almost consistent temperature changes and response times at ambient temperatures of 300 and 400 °C. For instance, the response time of G@NC60 dropped from ∼43.5 s at 200 °C to ∼11.6 s at ∼300 °C and 1.6 s at ∼ 400 °C. Meanwhile, the response time of G@NC75 was discovered slightly higher than that of G@NC60 at various environmental temperatures, and much lower than that of G@NC90, in that different NC contents corresponded to their own required amount of heat to absorb. It is noted that the electrical resistance of G@NC90 varied on the applied environmental temperatures, which indicates faster and greater resistance change with increasing ambient temperature. In addition, the response time of various NC content samples when directly attacked by an alcohol lamp flame is also presented in Figure 5d, whereas the rapid-fire alarm was triggered between 5 and 7 s approximately. As can be found below, Table 1 summarizes all collected data regarding the response temperature and time produced by the present sensor.

**Table 1:** Summary of Response Time and Response Temperature of Graphene/NC Composite Alarm Sensors

samples		G@NC60	G@NC75	G@NC90
response time at different environment temperature (s)	200 °C	43.5 ± 3.28	159.5 ± 4.23	
	300 °C	11.6 ± 2.31	12.5 ± 1.29	15.1 ± 1.59
	400 °C	1.6 ± 0.20	1.9 ± 0.21	4.4 ± 0.30
	flame	5.8 ± 0.31	6.2 ± 0.23	7.1 ± 0.68
response temperature at different heating rates (°C)	2.5 °C/s	174.3 ± 0.31	204.3 ± 0.46	231.8 ± 1.35
	5.0 °C/s	186.5 ± 2.03	209.2 ± 1.08	237.3 ± 1.45
	7.5 °C/s	192.7 ± 1.91	215.7 ± 2.48	246.8 ± 2.63

The relationship between temperature and resistance of the graphene/NC alarm sensors is further investigated for standardizing by TCR. Under ideal conditions, the theoretical and practical electrical conductivity of graphene is ∼185 000 and 200 000 cm^2^ V^–1^ s^–1^, respectively.^29^ TCR of graphene is not identically zero no matter if it is single-layer graphene or MLG. Suspended single-layer graphene has a small TCR because the dominant electron–electron scattering is dependent on temperature negligibly.^30^ However, the MLG utilized in this study has a larger TCR (negative value) because no electric field intensity can affect the carrier mobility in the interlayer channels.^27^ Testing under different environmental temperatures was conducted to observe the TCR of the alarm sensor with various NC content. TCR here is calculated using the following equation: . Table 2 shows the TCR of the graphene/NC composites within various temperature intervals ranging from 140 to 260 °C divided in every 20°. As the graphene/NC alarm sensors have various response temperatures with different NC contents, the electrical resistance of sensors may remain stable within the relatively lower temperature intervals. For instance, the TCR of G@NC75 and G@NC90 stay 0 before 200 and 220 °C, respectively. It is clearly observed that the TCR of each type of sample is close to ∼ −5% °C^–1^ within the temperature range of 200–220 °C, indicating that the main pyrolysis process of NC occurs in this temperature interval which is similar to the observed results from pure NC TGA tests. The TCR of G@NC60 and G@NC75 decreases to ∼ −2% °C^–1^ within the temperature range of 240–260 °C, which means that the decomposition process of NC and residue approached to finish in this range. Moreover, the resistance variation of G@NC90 in the range of 220–240 °C is similar to that of low NC content in the range of 200–220 °C, whereas the TCR of the same sensor is relatively stable at different heating rates. This phenomenon may indicate that there is a significant difference between the initial temperature at the beginning of pyrolysis of alarm sensors with high NC content and low NC content, but not between the whole NC thermal decomposition process. However, it still does not have a huge impact on the sensing mechanism of this temperature alarm sensor.

**Table 2:** TCR % Change per Celsius Degree of the Graphene/NC Composites within Various Temperature Intervals

		temperature range (°C)					
samples	heating rate (°C/min)	140–160 (%)	160–180 (%)	180–200 (%)	200–220 (%)	220–240 (%)	240–260 (%)
**NC60@G**	**2.5**	–4.59	–3.56	–4.63	–4.95	–3.05	–2.01
	**5**	0	–4.73	–3.84	–4.99	–4.03	–2.13
	**7.5**	0	–4.68	–2.91	–4.70	–4.96	–2.51
**NC75@G**	**2.5**	0	0	0	–5.00	–3.85	–2.53
	**5**	0	0	0	–5.00	–4.29	–2.09
	**7.5**	0	0	0	–4.98	–4.72	–2.03
**NC90@G**	**2.5**	0	0	0	0	–4.93	–3.99
	**5**	0	0	0	0	–4.87	–4.20
	**7.5**	0	0	0	0	–4.53	–4.13

The response temperature of graphene/NC alarm sensors is tunable by different NC contents within limits. To investigate the relationship between various NC contents and response temperature under different heating rates, the obtained response temperature data were analyzed utilizing linear fitting, as presented in Figure S6 (Supporting Information). First, the p-value achieved by two-way ANOVA without replication of NC contents and heating rates is 2.33 × 10^–5^ and 3.60 × 10^–3^, respectively, indicating that the association between these two factors and the response temperature was statistically significant. Moreover, the influence of NC content was relatively more effective. Obviously, the goodness of fit is higher than 0.98 at any heating rate, resulting in a reliable fitting degree and hypothesis accuracy. Therefore, the average value of estimated coefficients (1.8) can affirm the trend where each 1% increase in NC content leads to ∼1.8 °C increase in the response temperature. In addition, the two-way ANOVA with replication method was adopted to analyze the original response time data. The p-value has proven that the effects on response time from NC contents and environment temperature are also statistically significant when considering the integrated data (Table S2, Supporting Information).

### 3.6 Application Scenarios

Because the NC thermal decomposition reacts in instances and requires only heat (where oxygen is an indispensable factor), the alarm sensor is able to work stably in an oxygen-free or vacuum environment. The TGA results have proved that the graphene/NC composite reflects the same working process in a nitrogen environment as in normal ones. The extra tests were conducted to evaluate the operating state of the alarm sensor underwater. The testing setup and electrical resistance change curve are illustrated in Figure S4b and S7, respectively. The purpose of the underwater experiments of the G@NC75 in this paper is to prove the working principle of the graphene/NC alarm and expand the application in extreme environments. The most crucial factor which determines whether the alarm sensor can work underwater or not is the thermal degradation of the NC, rather than the effect of ambient temperature. No matter what the content of NC is 60, 75, or 90%, the adjustment of NC content will only affect the response temperature but not the working principle. The results showed that the sensor could still provide instant alarm signals, despite the heat absorption efficiency affected by water (Movie S4, Supporting Information). It follows then that the alarm sensor can work stably in other types of extreme environments (outdoor) in the form of paint or wallpaper sending high-temperature alarm signals to protect people’s lives. The excellent adhesion, mechanical robustness, and high flexibility of the graphene/NC film are also indispensable for high-performance alarm sensors in the form of wallpaper. It is well known that NC is one of the most versatile adhesives as a standard household cement. Comparison tests were carried out by other authors between annealed graphene/ethyl cellulose (EC) and graphene/NC films to investigate the adhesion and mechanical properties of graphene/NC films using ultrasonic treatment in water. Compared to the distinct delamination and breakage of graphene/EC film that occurred within 10 s in the ultrasonic bath, the graphene/NC film had negligible delamination and disintegration for over 1 min. However, the film on polyimides also showed even better performance in this test (up to 10 min), indicating this difference is based on adhesion at the interface between films and substrates. Furthermore, the peeling test was adopted to test the adhesion performance of the graphene/NC film on a glass slide. A piece of scotch tape was applied to the surface of graphene/NC film and then peeled at a consistent speed. The graphene/NC film showed excellent adhesion with glass because no evidence showed that visible residue was left on the tape. Compared to similar tests conducted on graphene/EC, which exhibits a noticeable change in graphene pattern. On the other hand, uncertainty and variation of the substrate may also affect the operating performance of the graphene/NC alarm sensor when it is utilized as wallpaper. However, according to previous studies, different substrates do not significantly affect the TCR of graphene.^27^ The substrate effect is lessened with the increasing thickness of graphene/NC film resulting in a stable electrical conductivity of graphene in annealed composites. Meanwhile, the evidence of the persistent alarm signal test and long-term stability test (see Figure S1, S3 and S8, Table S3, Movies S1, S2, and S3 in the Supporting Information) ensure the stability and flame retardancy of graphene as a wallpaper. For instance, G@NC75 film without a carrier burnt in 1.5 s, whereas it took another 0.5 s to burn up thoroughly when carried with the polyimide tape. However, G@NC75 with glass slide maintained its initial shape even with small bubble formation on the surface and did not catch fire in 10 s. Obviously, the flame retardancy of G@NC was superior when employed as wallpaper or coating materials. All types of graphene/NC alarm sensors maintained continuous danger alarm even after the samples were removed from a flame or specific temperature environment, demonstrating effective and stable fire warning that can be applied in various scenarios to detect high fire risk of combustible materials.

## 4 Conclusions

In summary, we report a fixed temperature fire alarm prepared using NC and MLG to reliably monitor early-fire risk of combustible materials. The graphene/NC alarm sensor remains in a state of electrical insulation though instantly turning conductive at high temperatures. Once encountering a flame attack, NC decomposes rapidly at high temperature and induces a distinct transition in its electrical resistance, causing the transformation process of the alarm sensor from being electrically insulated to an electron conductive state. Consequently, the LED alarm lamp connected to the graphene/NC sensor provides an ultrafast alarm response and early-fire warning signals under flame attacks or abnormal ambient high temperatures. The response temperature and time of graphene/NC alarm sensor keep similar upward tendencies as the embedded NC content increases. For example, the G@NC90 alarm remained stably insulated at an ambient temperature of 200 °C, resulting in a satisfactory responsive temperature (232 °C), instant response time (4.4 s), and sustained working time in the flame below the ignition temperature of most combustibles (400 °C). Furthermore, the response temperature and time of graphene/NC alarm sensor can be tuned by graphene/NC ratios to reduce fire risk of various combustible materials in different fire-prone scenarios and thus has promising applications in both indoor and outdoor environments. As the research results show, alarm sensors with lower NC contents (60 and 75%) reflected lower response temperature (161 and 208 °C) and faster response time, up to 1.6 and 1.9 s, respectively. Moreover, the graphene/NC composite film can be processed into various shapes due to its relatively high mechanical strength and flexibility. This developed fire alarm also broadens the applications of chemical-modified cellulose and graphene composites in temperature-induced resistance transition sensors with smart responsive behaviors, which provides a promising concept for its applications concerning public safety in terms of fire risk at high fire risk examinations.

## Supporting Information Available

Mechanical properties; analysis of the flame and water treatment snapshots; mechanism of electrical circuit in the process; ignition time and combustion speed with different type of carriers; EDS data comparison; analysis of stability after leaving the heat source; photographs of flame detection processes of flame rapid detection; and tables and figures that support the text (PDF)
Direct flame treatments of G@NC75 with the polyimide tape (MP4)
Direct flame treatments of G@NC75 with the polyimide tape (MP4)
Direct flame treatments of G@NC75 with the glass slide (MP4)
Combustion of G@NC75 tunable temperature sensor in the water with a heating element (MP4)
Combustion of G@NC60 tunable temperature sensor in the flame of the alcohol lamp (MP4)
Combustion of G@NC75 tunable temperature sensor in the flame of the alcohol lamp (MP4)
Combustion of G@NC90 tunable temperature sensor in the flame of the alcohol lamp (MP4)
