# Supplementary material for: Tunable Graphene/Nitrocellulose Temperature Alarm Sensors
Source: ACS Appl Mater Interfaces. 2022 Mar 11;14(11):13790–800. doi: 10.1021/acsami.2c02340 (PMC9098115; doi:10.1021/acsami.2c02340)
Supplement: Supplementary file 1 — am2c02340_si_001.pdf [file am2c02340_si_001.pdf]

Supporting Information

Tunable Graphene/Nitrocellulose Temperature Alarm Sensors

Wenyuan Wei[a], Yangpeiqi Yi[a], Jun Song[a], Xiaogang Chen[a], Jinhua Li[b], Jiashen Li[a]*

[a] Department of Materials, The University of Manchester, Manchester, M13 9PL, UK

[b] Hubei Key Laboratory of Polymer Materials, School of Materials Science and Engineering, Hubei University, Wuhan 430062, China

*Corresponding author. Tel: +44 (0) 161 306 5993. E-mail: Jiashen.li@manchester.ac.uk

Table S1. Tensile properties including the tensile strength, modulus and elongation at break of graphene-matrix composites sensors with different NC content

Sample	Tensile stress (MPa)	Modulus (GPa)	Elongation at break (%)
**G@NC60**	72.95±1.84	3.35±0.27	3.583±0.69
**G@NC75**	76.98±3.93	2.58±0.32	5.189±2.03
**G@NC90**	81.51±2.52	2.52±0.27	12.94±2.87
**Pure NC**	82.41±2.60	2.60±0.14	13.23±1.86

Table S2. P-value from statistical investigation of response time

	Scheme (a)	Scheme (b)
**Environment Temperatures**	$2.98E^{-22}$	$2.28E^{-13}$
**NC contents**	$2.38E^{-16}$	$2.14E^{-04}$
**Interaction**	$8.80E^{-19}$	0.436

Table S3. Ignition time and combustion speed for G@NC75 with different carrier. The data obtained from the Movie S1-S3

	Ignition time (s)	Combustion velocity (cm s^{-1})
**G@NC75**	0.067	4.412
**G@NC75 with Polyimide**	0.683	2.095
**G@NC75 with Glass side**	>10	/

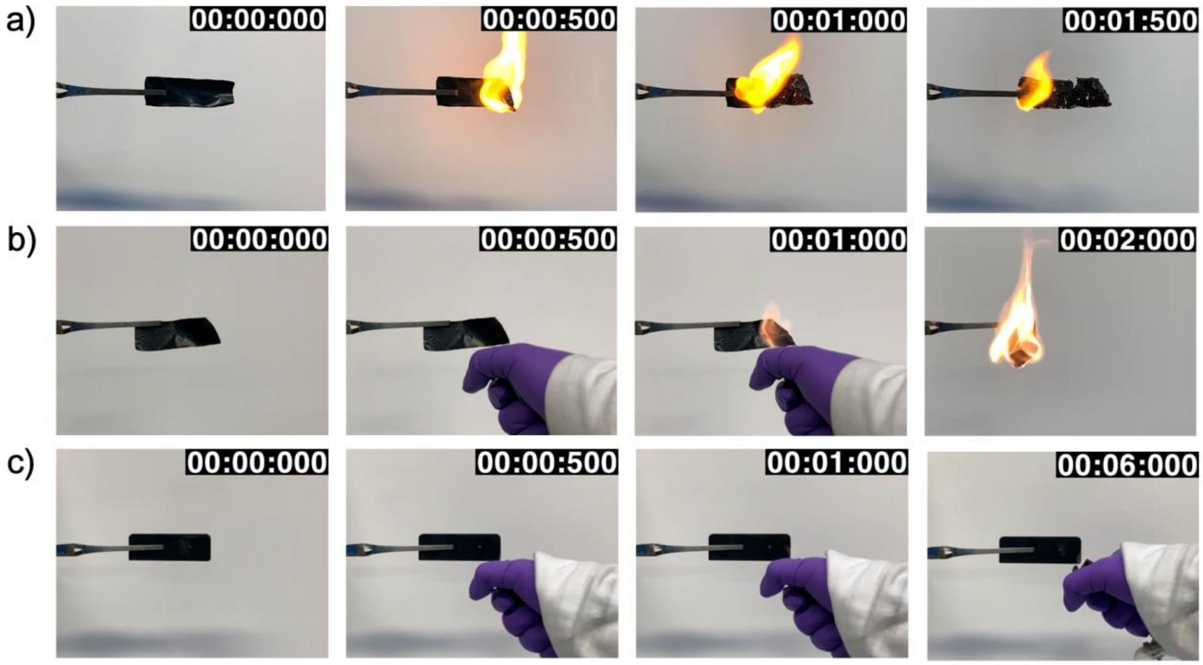

Figure S1. Snapshots of flame treatments of G@NC75: (a) pure G@NC75 (b) G@NC75 with Polyimide tape (c) G@NC75 with glass slide. The images were captured from the Movie S1, S2 and S3.

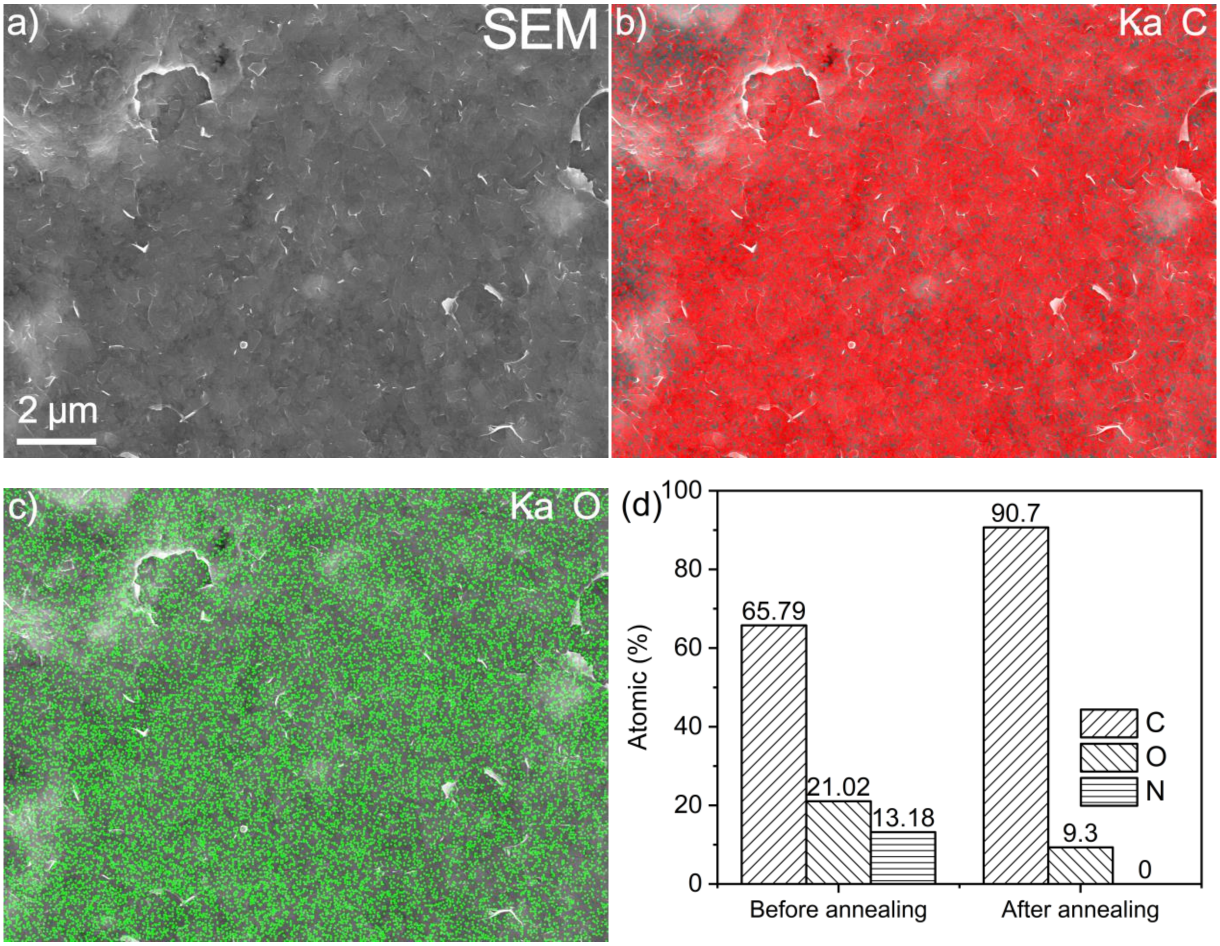

Figure S2. EDS data comparison of G@NC75.

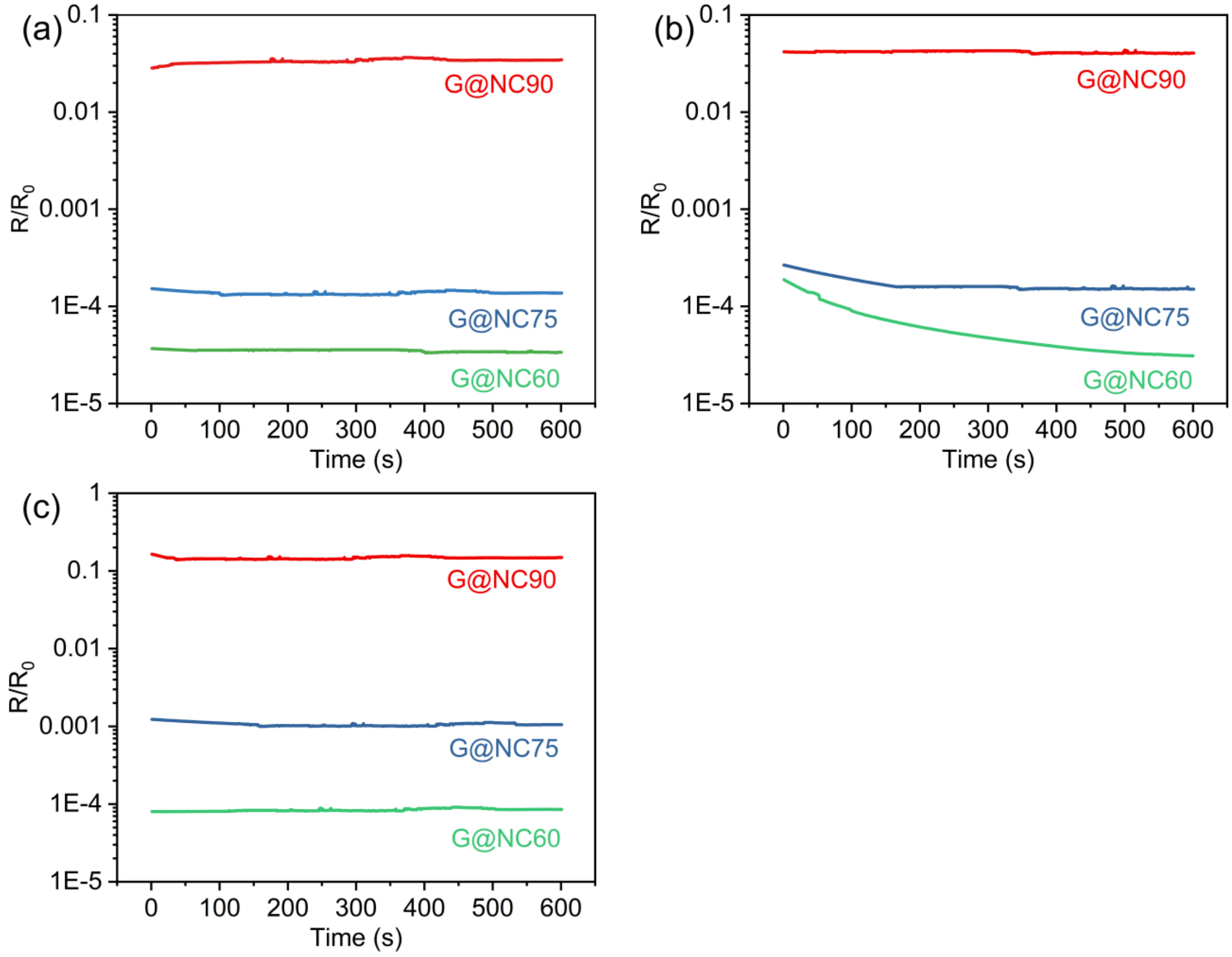

Figure S3. Electrical resistance changes of G@NCx turning off the heating oven after reaching 260 °C and then leaving for 600 seconds at various heating rate: a) 2.5 °C min^{-1}, b) 5.0 °C min^{-1}, c) 7.5 °C min^{-1}.

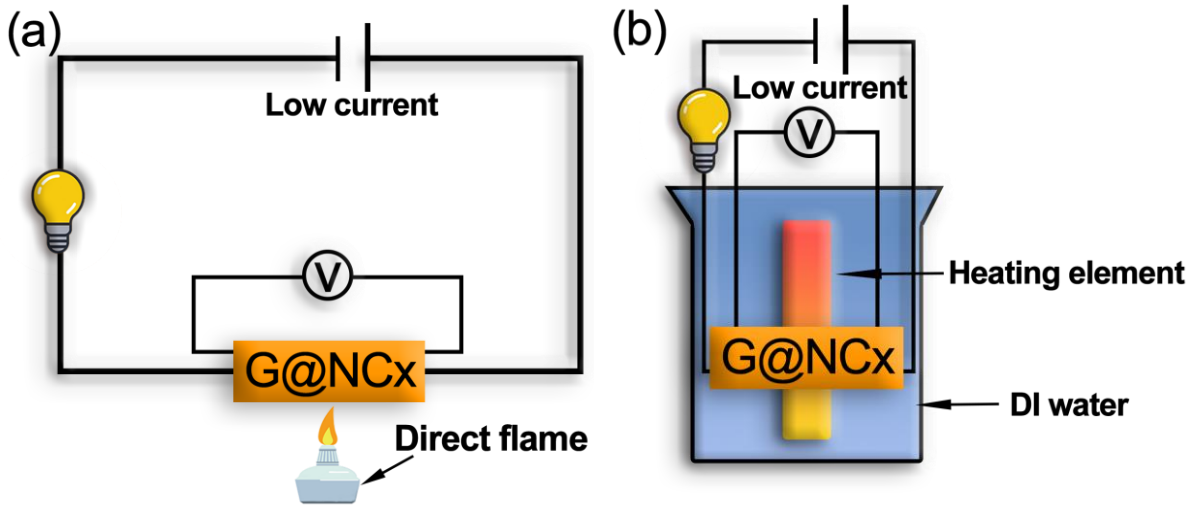

Figure S4. Schematic illustration of an electrical circuit for (a) the working process of the G@NCx temperature sensor in the flame. (b) the working process of the G@NCx temperature sensor in the water.

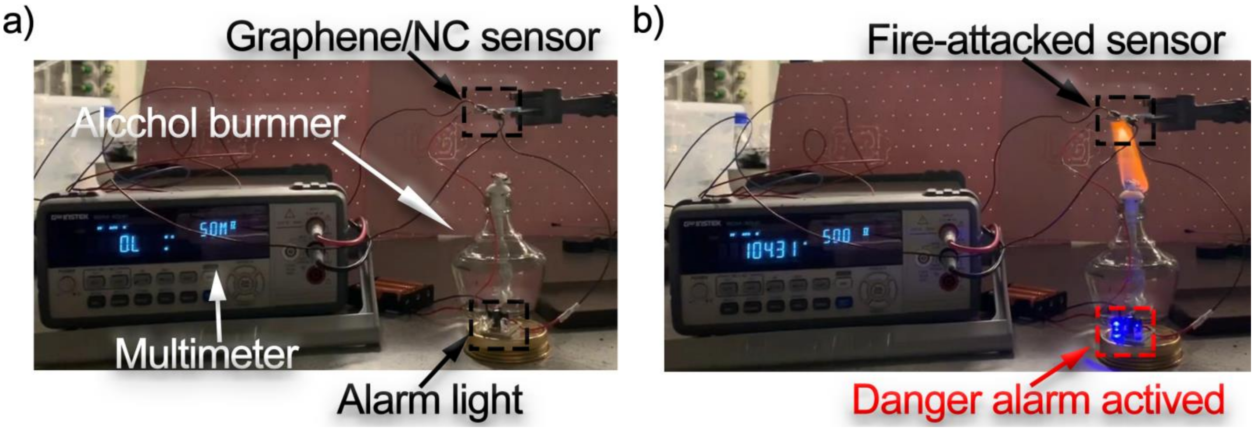

Figure S5. Photographs of flame detection processes of flame rapid detection and fire alarm of graphene/NC composites using the alcohol burner. (a) Testing set-up before fire-attack; (b) Testing set-up during fire-attack.

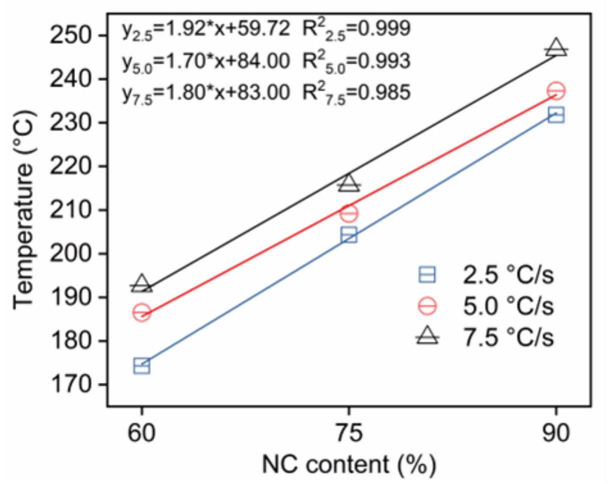

Figure S6. Linear relationship between response temperature and NC content.

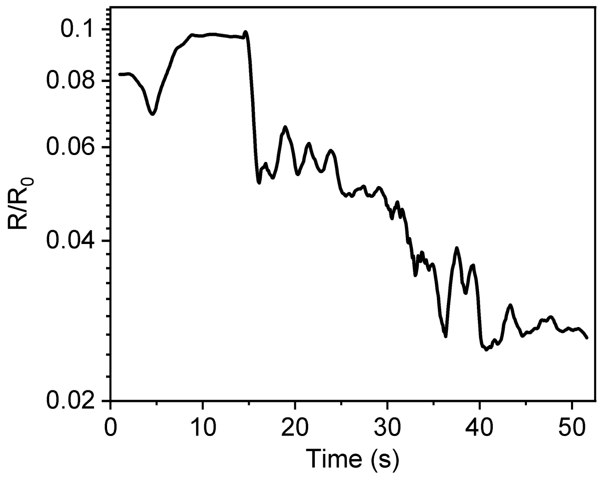

Figure S7. Electrical resistance changes of G@NC75 heated in the water environment from turning on the heating element.

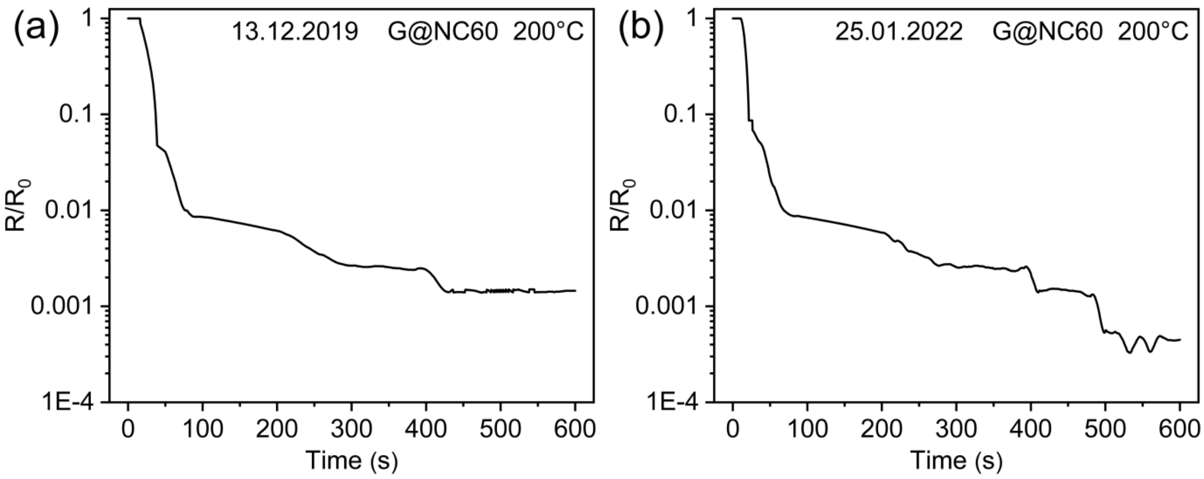

Figure S8. Electric resistance changes of G@NC60 sample under an environmental temperature of 200 °C observed in two years.

Movie Caption:

Movie S1. Direct flame treatments of G@NC75.

Movie S2. Direct flame treatments of G@NC75 with polyimide (PI) tape.

Movie S3. Direct flame treatments of G@NC75 with glass slide.

Movie S4. The combustion of G@NC75 tunable temperature sensor in the water with a heating element.

Movie S5. The combustion of G@NC60 tunable temperature sensor in the flame of the alcohol lamp.

Movie S6. The combustion of G@NC75 tunable temperature sensor in the flame of the alcohol lamp.

Movie S7. The combustion of G@NC90 tunable temperature sensor in the flame of the alcohol lamp.
